# Prediction of Atrial Fibrillation in Hospitalized Elderly Patients With Coronary Heart Disease and Type 2 Diabetes Mellitus Using Machine Learning: A Multicenter Retrospective Study

**DOI:** 10.3389/fpubh.2022.842104

**Published:** 2022-03-04

**Authors:** Qian Xu, Yan Peng, Juntao Tan, Wenlong Zhao, Meijie Yang, Jie Tian

**Affiliations:** ^1^College of Medical Informatics, Chongqing Medical University, Chongqing, China; ^2^Medical Data Science Academy, Chongqing Medical University, Chongqing, China; ^3^Collection Development Department of Library, Chongqing Medical University, Chongqing, China; ^4^Department of Cardiology, University-Town Hospital of Chongqing Medical University, Chongqing, China; ^5^Operation Management Office, Affiliated Banan Hospital of Chongqing Medical University, Chongqing, China; ^6^Department of Cardiology, Ministry of Education Key Laboratory of Child Development and Disorders, National Clinical Research Center for Child Health and Disorders, China International Science and Technology Cooperation Base of Child Development and Critical Disorders, Children's Hospital of Chongqing Medical University, Chongqing, China; ^7^Chongqing Key Laboratory of Pediatrics, Chongqing, China

**Keywords:** coronary heart disease, type 2 diabetes mellitus, atrial fibrillation, machine learning, prediction models

## Abstract

**Background:**

The objective of this study was to use machine learning algorithms to construct predictive models for atrial fibrillation (AF) in elderly patients with coronary heart disease (CHD) and type 2 diabetes mellitus (T2DM).

**Methods:**

The diagnosis and treatment data of elderly patients with CHD and T2DM, who were treated in four tertiary hospitals in Chongqing, China from 2015 to 2021, were collected. Five machine learning algorithms: logistic regression, logistic regression+least absolute shrinkage and selection operator, classified regression tree (CART), random forest (RF) and extreme gradient lifting (XGBoost) were used to construct the prediction models. The area under the receiver operating characteristic curve (AUC), sensitivity, specificity, and accuracy were used as the comparison measures between different models.

**Results:**

A total of 3,858 elderly patients with CHD and T2DM were included. In the internal validation cohort, XGBoost had the highest AUC (0.743) and sensitivity (0.833), and RF had the highest specificity (0.753) and accuracy (0.735). In the external verification, RF had the highest AUC (0.726) and sensitivity (0.686), and CART had the highest specificity (0.925) and accuracy (0.841). Total bilirubin, triglycerides and uric acid were the three most important predictors of AF.

**Conclusion:**

The risk prediction models of AF in elderly patients with CHD and T2DM based on machine learning algorithms had high diagnostic value. The prediction models constructed by RF and XGBoost were more effective. The results of this study can provide reference for the clinical prevention and treatment of AF.

## Introduction

Coronary heart disease (CHD) is the most common cardiovascular disease among the elderly (1). According to the data from the World Health Organization (2014), CHD is the main cause of death globally, accounting for more than 7 million deaths every year (2). As the most common type of diabetes mellitus (DM), type 2 diabetes mellitus (T2DM) has become the leading cause of morbidity and mortality worldwide. In 2019, there were 463 million individuals affected by diabetes globally (3). By 2040, the number of patients is expected to increase to 629 million, accounting for ~90% of all cases (4). T2DM is one of the most important complications of CHD. It is an important risk factor for the development of CHD and associated with the death of patients (5).

Atrial fibrillation (AF) is the most common arrhythmia, which is characterized by atrial activation disorder, that results in the deterioration of atrial function (6, 7). The prevalence of AF increased with age in recent studies. The overall prevalence was estimated to be 5.5%, rising from 0.7% in the 55–59 age group to 17.8% in the 85 years-and-above age group (8). Owing to the aging population, the prevalence of AF is expected to double by 2050 (9). AF in hospitalized elderly patients with CHD and T2DM is a very serious cardiac adverse event. Once patients develop AF, further thromboembolism will occur, leading to a high risk of disability and death (10, 11). Therefore, early identification and the timely management of AF in elderly patients with CHD and T2DM is very important. However, early detection of AF based on traditional indicators is difficult.

Therefore, this study is aimed at developing AF prediction models for the elderly patients with CHD and T2DM by using different machine learning algorithms to provide reference for the clinical prevention and treatment of AF. In order to the interpretability of results, the machine learning algorithms included in this study are mainly composed of logistic regression (LR), logistic regression+least absolute shrinkage and selection operator (LR+LASSO), classification and regression trees (CART), random forests (RF), and extreme gradient boosting (XGBoost).

## Methods

### Data Source

The study data were obtained from four tertiary hospitals in Chongqing, China, namely the Second Affiliated Hospital of Chongqing Medical University, the Third Affiliated Hospital of Chongqing Medical University, the University-Town Hospital of the Chongqing Medical University, and the Yong Chuan Hospital of the Chongqing Medical University. The transparent reporting of a multivariable predictive model for individual prognosis or diagnosis guidelines were followed for model development and validation. The clinical data were collected using electronic medical record systems. Data on patients with CHD and T2DM obtained for the period of 2015 to 2021 were used in the study.

The Ethics Committee of Chongqing Medical University approved the study. Written informed consent for participation was not required for this study owing to its retrospective design, and the study was undertaken in accordance with the national legislation and institutional requirements.

### Definition

The diagnosis of AF (ICD-10, I48) was based on AHA/ACC/ESC 2006 atrial fibrillation guidelines (12). Two physicians with rich clinical experience were convened to diagnose together with echocardiography, electrocardiogram, and laboratory examinations.

### Inclusion and Exclusion Criteria

Inclusion criteria comprised the following: (i) data obtained from 2015 to 2021, (ii) patients aged ≥ 65 years, and (iii) hospitalization (s) for CHD and T2DM.

**Figure 1:**
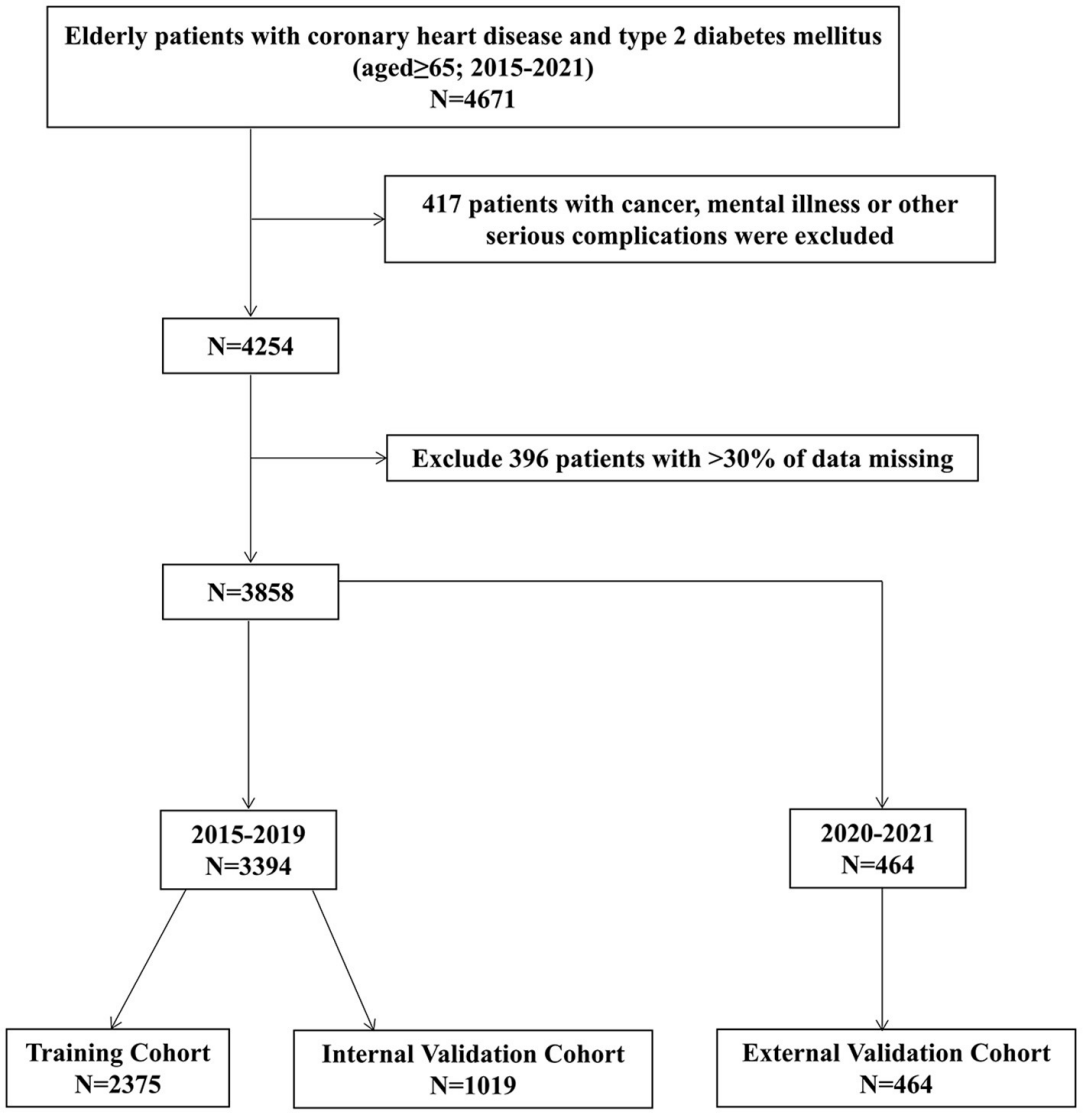
Flow of inclusions and exclusions.

Exclusion criteria comprised the following: (i) patients with cancer, mental illness, or other serious complications and (ii) patients with >30% of missing data. The study selection process is depicted in the following flow chart (Figure 1).

### Data Collection

Based on previous studies, 28 possible risk factors for predicting AF were selected, namely age, gender, smoking status, drinking status, hypertension, diastolic blood pressure (DBP), γ-glutamyltransferase (GGT), white blood cell (WBC), neutrophil-to-lymphocyte ratio (NLR), lymphocyte-to-monocyte ratio (LMR), platelet-lymphocyte (PLR), blood creatinine, total bilirubin (TB), uric acid (UA), urea Nitrogen (UN), low-density lipoprotein (LDL), hemoglobin, alanine aminotransferase (ALT), albumin (ALB), triglycerides (TGs), total cholesterol (TC), high-density lipoprotein (HDL), glycated hemoglobin (HbA1c), alkaline phosphatase (ALP), aspartate aminotransferase (AST), blood potassium, blood calcium, and blood phosphorus.

### Statistical Analyses

The multiple imputation method (predictive mean matching) was used to fill in the missing continuous variables. Since the visit time period of the patients included in this study spanned from 2015 to 2021, this study can further subjected to temporal validation (13, 14). Therefore, the data included in this study were divided into two parts: 2015 to 2019 and 2020 to 2021 according to date. The data from 2015 to 2019 were divided into the training cohort and internal validation cohort based on the ratio of 7:3. The data from 2020 to 2021 were used as an external validation cohort. The training cohort was used to develop predictive models using machine learning (CART, RF, and XGBoost) and logistic regression algorithms, and then the final performance of each model was verified and compared in the internal validation cohort and external validation cohort. The area under the receiver operating characteristic curve (AUC), sensitivity, specificity, and accuracy were used as the comparison measures between different models.

Continuous variables were expressed as mean ± standard deviation (SD) or median (interquartile range), according to the distribution of normality. Categorical variables were reported as counts with percentages. Continuous variables were tested by *t*-test or Mann-Whitney *U*-test whereas categorical variables were analyzed by Chi-square test (χ^2^-test). All statistical analyses used two-sided tests, and *P* < 0.05 was considered statistically significant. Statistical analyses were performed using SPSS 22.0 and R (version 4.0.2, Vienna, Austria).

## Results

### Patient Characteristics

A total of 3,858 patients with CHD and T2DM were included in this study. They were divided into a training cohort (*N* = 2,375), an internal validation cohort (*N* = 1,019), and an external validation cohort (*N* = 464). The Mann-Whitney *U*-test showed that there was no significant difference before and after interpolation (Table 1).

**Table 1:** Comparison of continuous variables before and after interpolation.

**Variables**	**Before interpolation**	**After interpolation**	***P-*values**
DBP (IQR, mmHg)	77.00 (69.00, 85.00)	77.00 (69.00, 85.00)	0.894
GGT (IQR, IU/L)	26.00 (18.00, 44.00)	26.00 (18.00, 44.67)	0.760
WBC (IQR, × 109/L)	6.78 (5.52, 8.56)	6.78 (5.52, 8.56)	1.000
NLR (IQR)	3.33 (2.32, 5.51)	3.33 (2.32, 5.52)	0.969
LMR (IQR)	3.63 (2.37, 5.23)	3.63 (2.37, 5.23)	0.999
Blood creatinine	73.35 (57.70, 97.73)	73.35 (57.70, 97.78)	0.981
TB (IQR, umol/l)	10.30 (7.60, 14.00)	10.30 (7.60, 14.00)	0.944
UA (IQR, umol/L)	339.30 (273.48, 417.03)	338.90 (273.30, 417.08)	0.962
UN (IQR, mmol/L)	6.54 (5.20, 8.58)	6.54 (5.20, 8.57)	0.973
LDL (IQR, mmol/L)	2.23 (1.67, 2.91)	2.24 (1.67, 2.91)	0.871
Hemoglobin (IQR, g/L)	127.00 (115.00, 138.00)	127.00 (115.00, 138.00)	1.000
PLR (IQR)	128.43 (94.69, 178.29)	127.98 (94.40, 177.71)	0.776
ALT (IQR, IU/L)	18.16 (13.00, 27.14)	18.12 (13.00, 27.58)	0.987
ALB (IQR, g/L)	39.50 (36.50, 42.60)	39.50 (36.50, 42.60)	0.966
TGs (IQR, mmol/L)	1.41 (1.02, 2.02)	1.42 (1.02, 2.03)	0.774
TC (IQR, mmol/L)	4.10 (3.37, 4.96)	4.09 (3.37, 4.96)	0.958
HDL (IQR, mmol/L)	1.10 (0.91, 1.33)	1.09 (0.91, 1.32)	0.587
HbA1c (IQR, %)	7.41 (6.60, 9.04)	7.44 (6.60, 9.10)	0.743
ALP (IQR, IU/L)	74.00 (61.00, 92.00)	74.00 (61.00, 92.00)	0.878
AST (IQR, IU/L)	20.20 (16.00, 27.00)	20.20 (16.01, 27.00)	0.968
Blood potassium (IQR, mmol/L)	4.02 (3.72, 4.34)	4.02 (3.71, 4.33)	0.639
Blood calcium (IQR, mmol/L)	2.23 (2.13, 2.33)	2.23 (2.13, 2.33)	0.921
Blood phosphorus (IQR, mmol/L)	1.09 (0.95, 1.24)	1.09 (0.94, 1.23)	0.160

*DBP, diastolic blood pressure; GGT, γ-glutamyltransferase; WBC, white blood cell; NLR, neutrophil-to-lymphocyte ratio; LMR, lymphocyte-to-monocyte ratio; TB, total bilirubin; UA, uric acid; UN, urea nitrogen; LDL, low-density lipoprotein; PLR, platelet-lymphocyte ratio; ALT, alanine aminotransferase; ALB, albumin; TGs, triglycerides; TC, total cholesterol; HDL, high-density lipoprotein; HbA1c, glycated hemoglobin; ALP, alkaline phosphatase; AST, aspartate aminotransferase; IQR, interquartile range*.

In the training cohort, the results showed that in comparison with patients in the non-AF group, patients who suffered from AF had a higher age (*P* < 0.001), higher DBP (*P* = 0.005), GGT (*P* < 0.001), NLR (*P* < 0.001), blood creatinine (*P* = 0.001), higher TB (*P* < 0.001), UA (*P* < 0.001), UN (*P* = 0.001), and AST (*P* < 0.001). The levels of LMR (*P* < 0.001), LDL (*P* < 0.001), ALB (*P* < 0.001), TGs (*P* < 0.001), TC (*P* < 0.001), HDL (*P* = 0.007), and blood calcium (*P* < 0.001) in the AF group are lower compared with those in the non-AF group (Table 2).

**Table 2:** Univariate analyses of variables associated with AF.

**Variables**	**AF (*N* = 270)**	**Non-AF (*N* = 2,105)**	***P-*values**
Gender (*n*, %)	119 (44.07%)	924 (43.90%)	0.999
Smoking status (*n*, %)	78 (28.89%)	574 (27.27%)	0.625
Drinking status (*n*, %)	55 (20.37%)	411 (19.52%)	0.804
Hypertension (*n*, %)	206 (76.30%)	1,670 (79.33%)	0.283
Age (IQR, years)	78.00 (73.00, 83.00)	75.00 (70.00, 81.00)	<0.001
DBP (IQR, mmHg)	79.00 (71.00, 88.00)	77.00 (69.00, 85.00)	0.005
GGT (IQR, IU/L)	36.00 (22.55, 69.00)	25.00 (17.60, 43.00)	<0.001
WBC (IQR, × 109/L)	6.82 (5.57, 8.39)	6.73 (5.53, 8.50)	0.848
NLR (IQR)	4.44 (2.74, 6.36)	3.23 (2.28, 5.29)	<0.001
LMR (IQR)	3.18 (2.02, 4.69)	3.81 (2.46, 5.44)	<0.001
Blood creatinine	80.30 (62.78, 105.18)	72.40 (56.90, 97.20)	0.001
TB (IQR, umol/l)	12.30 (9.20, 17.55)	10.00 (7.40, 13.70)	<0.001
UA (IQR, umol/L)	370.40 (296.43, 469.68)	332.10 (270.40, 410.00)	<0.001
UN (IQR, mmol/L)	7.09 (5.41, 9.46)	6.51 (5.23, 8.39)	0.001
LDL (IQR, mmol/L)	1.92 (1.47, 2.62)	2.27 (1.69, 2.95)	<0.001
Hemoglobin (IQR, g/L)	125.00 (112.25, 137.75)	127.00 (115.00, 139.00)	0.377
PLR (IQR)	129.96 (95.24, 180.14)	127.95 (94.27, 175.86)	0.356
ALT (IQR, IU/L)	19.00 (12.02, 28.00)	18.00 (13.00, 27.00)	0.722
ALB (IQR, g/L)	38.40 (35.79, 41.00)	39.60 (36.60, 42.70)	<0.001
TGs (IQR, mmol/L)	1.17 (0.83, 1.72)	1.42 (1.04, 2.03)	<0.001
TC (IQR, mmol/L)	3.64 (3.02, 4.54)	4.16 (3.41, 4.97)	<0.001
HDL (IQR, mmol/L)	1.05 (0.85, 1.27)	1.10 (0.92, 1.32)	0.007
HbA1c (IQR, %)	7.35 (6.50, 8.50)	7.40 (6.60, 9.00)	0.129
ALP (IQR, IU/L)	74.80 (58.43, 96.08)	74.00 (61.00, 91.90)	0.579
AST (IQR, IU/L)	22.17 (17.25, 31.23)	20.00 (16.00, 27.00)	0.001
Blood potassium (IQR, mmol/L)	3.97 (3.69, 4.36)	4.02 (3.72, 4.32)	0.694
Blood calcium (IQR, mmol/L)	2.21 (2.11, 2.29)	2.24 (2.14, 2.34)	0.001
Blood phosphorus (IQR, mmol/L)	1.09 (0.96, 1.25)	1.10 (0.95, 1.24)	0.942

*AF, atrial fibrillation; DBP, diastolic blood pressure; GGT, γ-glutamyltransferase; WBC, white blood cell; NLR, neutrophil-to-lymphocyte ratio; LMR, lymphocyte-to-monocyte ratio; TB, total bilirubin; UA, uric acid; UN, urea nitrogen; LDL, low-density lipoprotein; PLR, platelet-lymphocyte ratio; ALT, alanine aminotransferase; ALB, albumin; TGs, triglycerides; TC, total cholesterol; HDL, high-density lipoprotein; HbA1c, glycated hemoglobin; ALP, alkaline phosphatase; AST, aspartate aminotransferase; IQR, interquartile range*.

### Predictive Effects of Different Models

**Figure 2:**
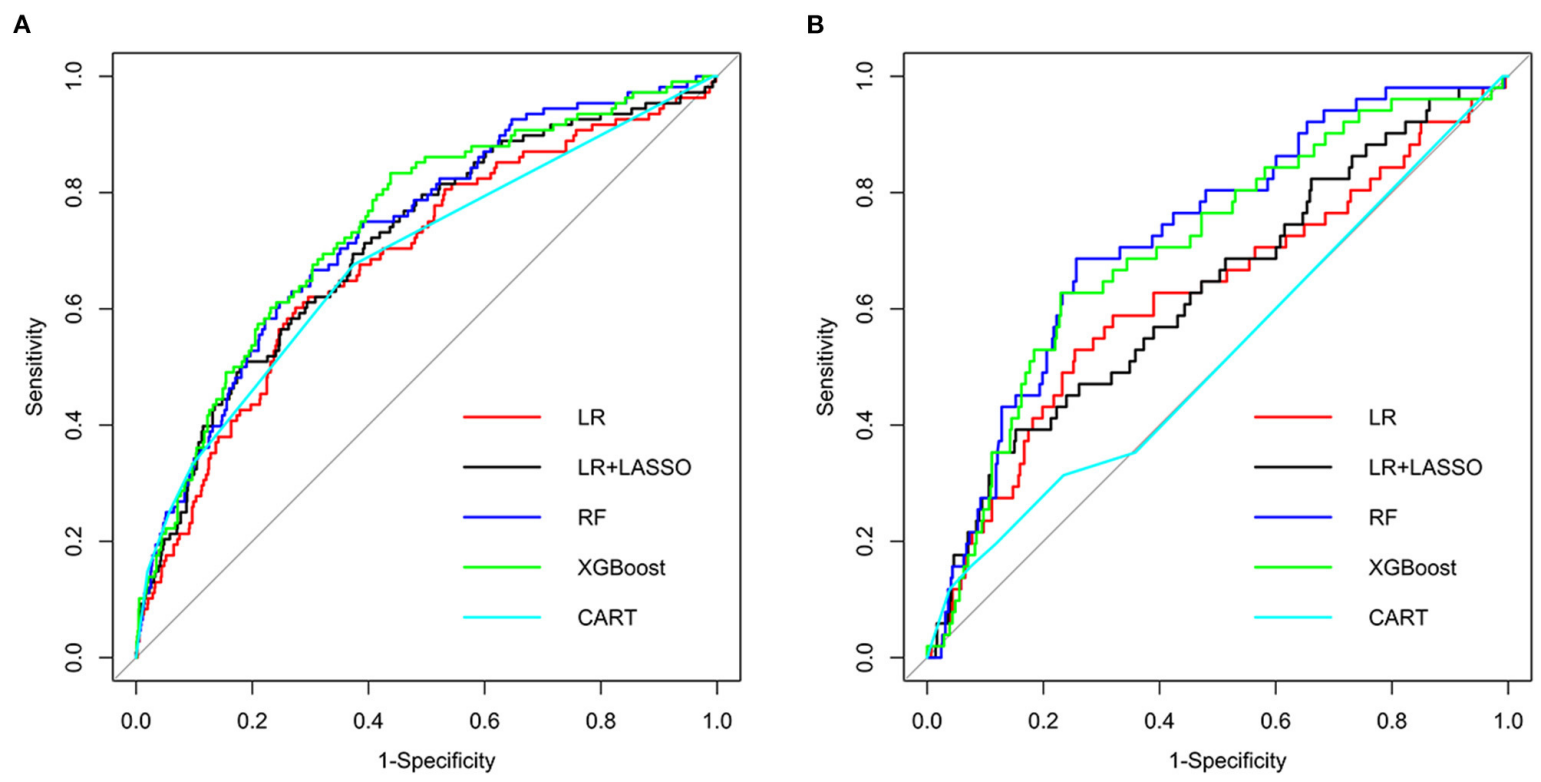
Receiver operating characteristic (ROC) curves of five different models in internal validation cohort **(A)** and external validation cohort **(B)**.

Five models were generated, including LR, LR+LASSO, CART, RF, and XGBoost, to predict the development of AF in elderly patients with CHD and T2DM after admission. Figure 2 shows the performance of the five different models in predicting AF in the internal validation cohort and external validation cohort in terms of ROC curves. In the internal validation cohort, AUC shows that the XGBoost model has the best prediction effectiveness on AF, and AUC was 0.743 compared with other models. In the external validation cohort, the highest AUC model was RF.

Tables 3, 4, respectively, show the detailed performance indicators of the five models in the internal verification cohort and external verification cohort. In the internal validation cohort, XGBoost had the highest AUC (0.743) and sensitivity (0.833), and RF had the highest specificity (0.753) and accuracy (0.735). In the external verification, RF had the highest AUC (0.726) and sensitivity (0.686), and CART had the highest specificity (0.925) and accuracy (0.841).

**Table 3:** Detailed performance metrics for the five models in internal validation.

**Model**	**Sensitivity**	**Specificity**	**Accuracy**	**AUC (95%CI)**
LR	0.602	0.726	0.712	0.684 (0.629–0.739)
LR+LASSO	0.694	0.627	0.633	0.712 (0.659–0.765)
CART	0.676	0.626	0.631	0.686 (0.632–0.739)
RF	0.611	0.753	0.735	0.733 (0.683–0.783)
XGBoost	0.833	0.562	0.587	0.743 (0.693–0.792)

*LR, logistic regression; CART, Classification and Regression Tree; RF, random forest; AUC, area under the receiver operating characteristic curve; CI, confidence interval*.

**Table 4:** Detailed performance metrics for the five models in external validation.

**Model**	**Sensitivity**	**Specificity**	**Accuracy**	**AUC (95%CI)**
LR	0.529	0.746	0.722	0.621 (0.532–0.711)
LR+LASSO	0.353	0.889	0.828	0.630 (0.659–0.765)
CART	0.157	0.925	0.841	0.523 (0.444–0.601)
RF	0.686	0.743	0.733	0.726 (0.655–0.797)
XGBoost	0.627	0.770	0.754	0.705 (0.628–0.781)

*LR, logistic regression; CART, Classification and Regression Tree; RF, random forest; AUC, area under the receiver operating characteristic curve; CI, confidence interval*.

**Figure 3:**
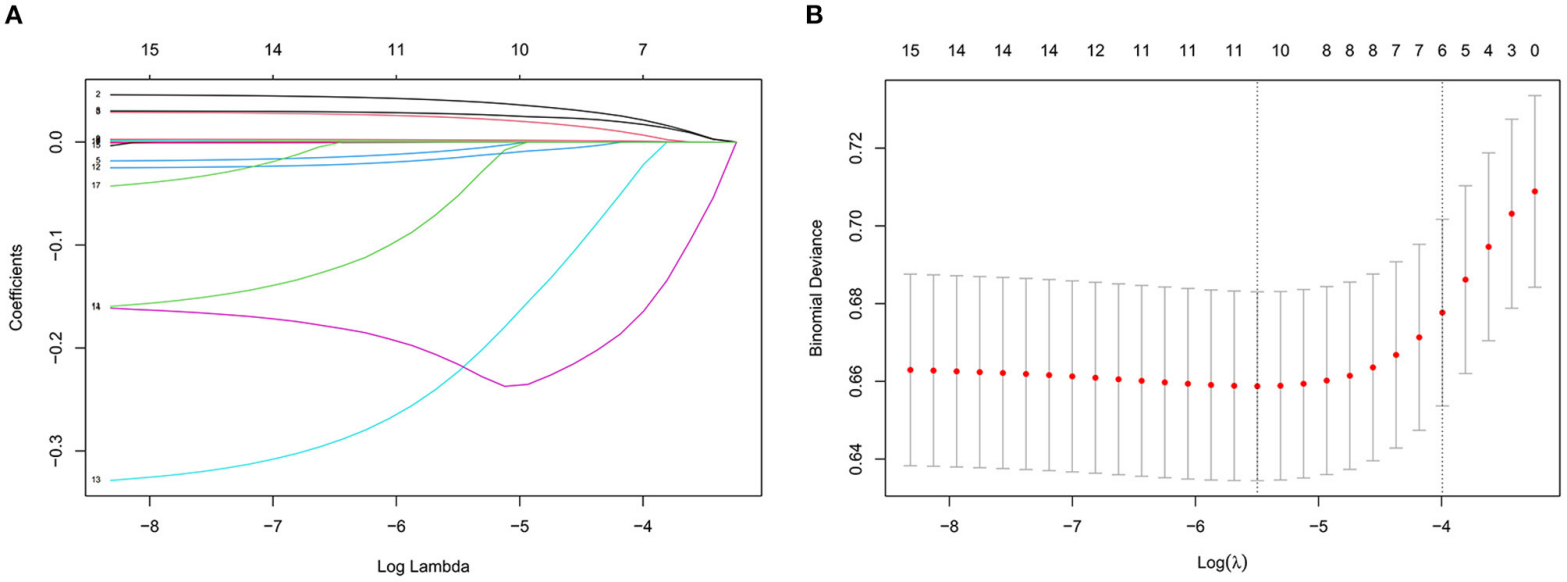
Features selection by LASSO. **(A)** LASSO coefficients profiles (y-axis) of the 16 features. The upper x-axis is the average numbers of predictors and the lower x-axis is the log (λ). **(B)** Five-fold cross-validation for tuning parameter selection in the LASSO model.

**Figure 4:**
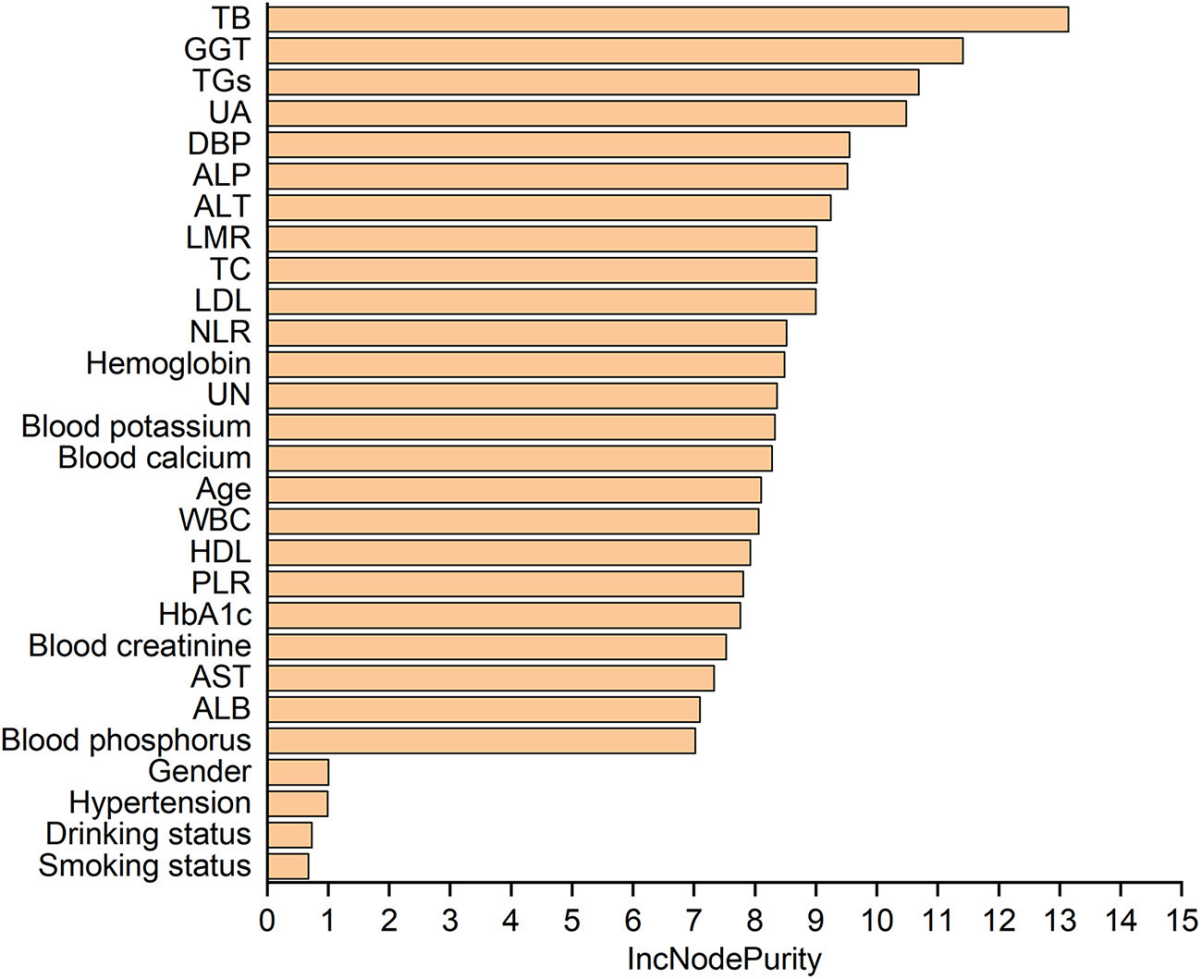
Importance analysis of indexes in RF model.

**Figure 5:**
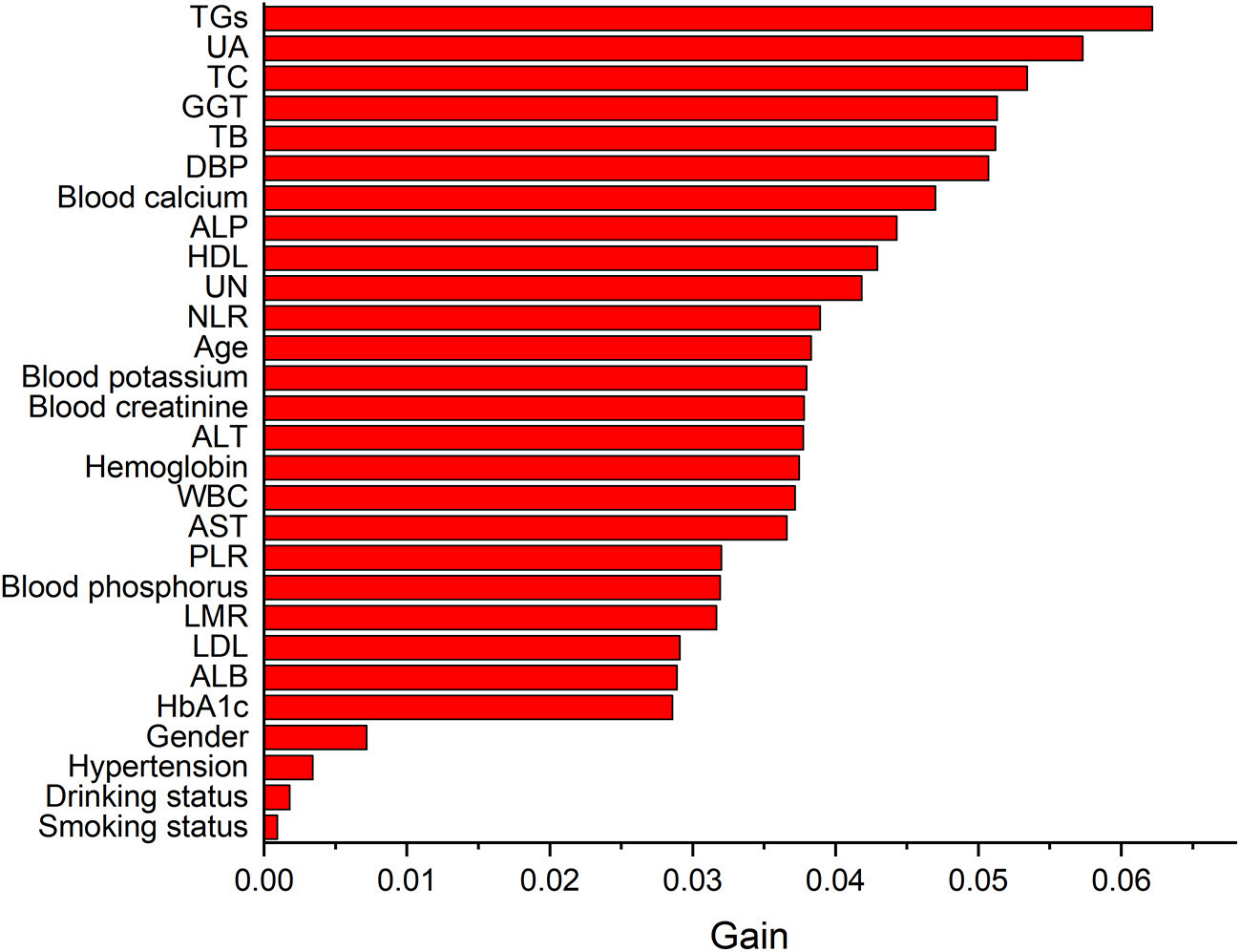
Importance analysis of indexes in XGBoost model.

Table 5 lists the importance levels of the top five features in the top three AUC models in both the internal validation cohort and the external validation cohort. As shown in Table 5, TB, TGs and UA were the top three features that promoted the development of the prediction models for AF in coronary heart disease and type 2 diabetes mellitus patients. Figure 3 shows the feature screening process of LR+LASSO model, Figures 4, 5 shows the feature importance ranking of the RF model and the XGBoost model, respectively.

**Table 5:** Ranks of feature importance in RF, XGBoost, and LR+LASSO for predicting AF.

**Rank**	**RF**	**XGBoost**	**LR+LASSO**
1	TB	TGs	TC
2	GGT	UA	TGs
3	TGs	TC	Age
4	UA	GGT	TB
5	DBP	TB	UA

*LR, logistic regression; RF, random forest; DBP, diastolic blood pressure; GGT, γ-glutamyltransferase; TB, total bilirubin; UA, uric acid; TGs, triglycerides; TC, total cholesterol*.

## Discussion

In this study, A total of 3,858 patients with CHD and T2DM were included, and then 28 possible risk factors were selected for predicting AF. Five machine learning algorithms were used to construct the prediction models, and then the final performance of each model was verified and compared in the internal validation cohort and the external validation cohort. The results showed that the prediction models constructed by RF and XGBoost were more effective (delong test showed that there was no significant difference between the two models, *P* = 0.654). TB, TGs and UA were the three most important predictors of AF.

Machine learning algorithms could deeply mine and analyze big data, and it had been widely used in disease predictions and prognostication (15–17). RF is one of the widely used algorithms in machine learning. It uses a set model of various decision trees (18). RF rely on computers to learn all of the complex non-linear interactions between variables by minimizing the error between observation results and prediction results (19), and it uses bootstrap aggregation and randomization of predictors to obtain high disease prediction accuracy (20, 21). In addition, XGBoost is also an efficient and widely used machine learning method (22). It is an integrated algorithm belonging to a class of boosting algorithms. The core of the boosting algorithm is to integrate many weak classifiers to form a strong classifier, so as to improve the accuracy of classification. XGBoost helps to reduce overfitting compared to gradient tree boosting by only a random subset of descriptors in building a tree and is known as the “regularized boosting” technique (23).

Our study shows that high TB was a risk factor for AF in elderly patients with CHD and T2DM. Recently, studies found that TB levels are related to CVD and AF (24, 25). Bilirubin is a product of hemoglobin catabolism and has long been used as a diagnostic indicator for hepatobiliary diseases and hemolytic diseases (26). AF promotes the occurrence of inflammation and the increase of inflammatory markers, while TB, as an antioxidant stress factor, increases compensatory in order to balance oxidation and antioxidation in the body (27). Therefore, for elderly patients with CHD and T2DM, when TB level is significantly increased, there is risk of AF.

TGs are the most abundant lipids in the human body. Most tissues can use TG decomposition products to supply energy. At the same time, liver, fat, and other tissues can also synthesize TGs and store them in adipose tissue (28, 29). TGs have been proven to be a risk factor for cardiovascular disease (CVD), but its role in the development of AF in the elderly is unclear (30). The study found that low TGs were a risk factor for AF in elderly patients with CHD and T2DM. A cross-sectional survey showed that Low TGs may be a marker of CVD risk in Chinese patients with long-term T2DM (31), which is consistent with the results of our study. Of note, clinicians usually pay attention to the risk caused by elevated TGs and ignore the impact of low TGs in patients, especially in elderly patients with CHD and T2DM. The results of this study suggest that clinicians should pay attention to the TG level of elderly patients with CHD and T2DM in order to prevent atrial fibrillation in advance.

This study also showed that high UA was a risk factor for AF in elderly patients with CHD and T2DM. In recent years, several cross-sectional studies and prospective studies had shown that a high UA level is a potential risk factor for AF (32–34). Another study showed that high UA is associated with an increased prevalence of AF in hospitalized patients with T2DM, independent of multiple risk factors and potential confounders (35). Yutong Ji et al. also found that there is an association between AF and high UA in elderly patients, high UA is associated with persistent or permanent AF (36). Therefore, reducing the level of UA can reduce the risk of AF in elderly patients to a certain extent.

This study is the first to investigate the predictive model for AF in hospitalized elderly patients with coronary heart disease and type 2 diabetes mellitus. However, this study also has several limitations. Firstly, selection bias may exist due to the retrospective nature of the investigation and the imbalanced dataset. However, a multicenter and relatively large training cohort were used to build models, which was further subjected to temporal validation. Secondly, data on social support, socioeconomic status, and some other important factors were not available. Further research is warranted to explore the impact of these important indicators.

In conclusion, compared with classic logistic regression models, machine learning models (XGBoost and RF) have better performance in predicting AF in elderly patients with CHD and T2DM using features that were easily available at admission. Predictive models using machine learning algorithms can help clinicians predict AF early and implement targeted treatment measures.

## Data Availability Statement

The raw data supporting the conclusions of this article will be made available by the authors, without undue reservation.

## Ethics Statement

The Ethics Committee of Chongqing Medical University approved the study. Written informed consent for participation was not required for this study due to its retrospective design and the study was undertaken in accordance with national legislation and institutional requirements.

## Author Contributions

QX, YP, JTa, and MY: conception, methodology, and draft of the manuscript. WZ: suggestions for amendment. JTi: review and supervision. All authors contributed to the article and approved the submitted version.

## Funding

This research was supported by Chongqing Federation of Social Sciences (No. 2021NDQN65) and the Intelligent Medical Foundation of Chongqing Medical University (No. ZHYX202029).

## Conflict of Interest

The authors declare that the research was conducted in the absence of any commercial or financial relationships that could be construed as a potential conflict of interest.

## Publisher's Note

All claims expressed in this article are solely those of the authors and do not necessarily represent those of their affiliated organizations, or those of the publisher, the editors and the reviewers. Any product that may be evaluated in this article, or claim that may be made by its manufacturer, is not guaranteed or endorsed by the publisher.
